# Position specific peak running demands, and influence of bout type in professional rugby union

**DOI:** 10.1371/journal.pone.0320286

**Published:** 2025-04-04

**Authors:** Daniel J. Glassbrook, Lewis D. Williams, Kevin J. McShane

**Affiliations:** 1 Newcastle Falcons Rugby Football Club, Newcastle upon Tyne, United Kingdom; 2 Wolfson Research Institute for Health and Wellbeing, Durham University, Durham, United Kingdom; 3 Department of Sport and Exercise Sciences, Faculty of Social Sciences and Health, Durham University, Durham, United Kingdom; Technological University Dublin—Tallaght Campus, IRELAND

## Abstract

**Objectives:**

To quantify peak running intensity in professional rugby union across position groups, and peak running intensity differences between bout types (i.e., whole, starter, substitute).

**Design:**

Longitudinal study.

**Method:**

Global positioning systems were used to assess the activity of 36 professional rugby union players. A moving average approach was used to identify the 1- to 10-minute peak intensity period distances, and time spent above 80% and 90% of individual 1-minute match peak. Differences between position groups and bout type were determined by magnitude-based inferences.

**Results:**

All position groups showed *most likely moderate* to *most likely large* differences in peak intensity periods, except for tight 5 vs. backrow (*possibly trivial small* and *possibly small*), and half-backs vs. outside backs (*very likely trivial small* to *likely trivial small*). No position group comparison for time spent above 80% and 90% of 1-minute match peak resulted in *moderate* or greater differences. *Possibly moderate* to *most likely moderate* difference were observed between forwards whole vs. forward substitutes in 2- to 10-minute peak periods; *most likely moderate* differences were observed between forwards starters vs. forward substitutes in 10-minute peak intensity period; and *most likely moderate* differences were observed between backs whole vs. backs substitutes in the 1-minute peak intensity period. For time spent above 80% and 90% of 1-minute match peak all bout type comparisons resulted in *most likely moderate* to *most likely large* differences.

**Conclusions:**

There are meaningful differences between position groups in peak running intensity in professional rugby union, and substitute players perform lower peak intensity running than whole or starters.

## Introduction

Rugby union is a high-intensity team sport, and is physical in nature and philosophy [1,2], with players required to be competitive in a range of capacities, such as: aerobic fitness, speed, strength, and power [1,3]. Matches are comprised of short bouts (5- to 15-seconds) of maximal or high intensity activity such as: accelerating, sprinting and contact situations (e.g., the tackle, rucks, and scrums) and low intensity activity of longer durations ( ≤ 40-seconds) such as: standing, walking or jogging [4]. Professional rugby union matches are comprised of two 40-minute halves, separated by a half time break of no longer than 15-minutes. There are 15 players on each team; the starting team for each side is comprised of forwards (numbers 1–8; props, hooker, locks, flankers, and number 8) and backs (numbers 8–15; scrum-half, fly-half, inside centre, outside centre, wings, and fullback). There are then also eight substitute players available to coaches to be included as tactical substitutions [5], whereby coaches are able to select larger, more physically competitive players to start matches, then replace them with fresh players when those starters fatigue [5,6]. Substitute players may also be activated to replace an injured player. Although substitute players will typically have reduced loads than starting or whole game players due to reduced playing time [7], it is possible that running intensity may be larger in substitute players, as there is less requirement to pace themselves [6,8]. Prior research has shown generally improved running performance in substitutes than starters or whole game players in both forwards and backs in relative distance (m·min^ − 1^), and high-intensity distance (m·min^ − 1^ > 5 m·s^ − 1^), however these were only *small* differences (Effect Size (ES): 0.2–0.5) [9]. Other research has shown significantly greater high speed distance (m·min^ − 1^; > 4 m·s^ − 1^; v*ery likely* medium ES: 0.95), and acceleration frequency (min per acceleration; *very likely large* ES: 1.32) in substitute forward players than forward starters [8]. Substitute forward players also showed significantly greater high speed distance (*very likely large* ES: 1.47) and acceleration frequency (v*ery likely large* ES: 1.39) than whole game forward players [8]. No significant results were shown in the backs position groups, regardless of bout type.

Quantifying the running workload and intensity of match-play is important for high-performance coaches and assists in the prescription and monitoring of training to replicate the demands of match-play [10,11]. The locomotor demands of rugby union match-play are typically measured by Global Positioning Systems (GPS), with workloads characterised by distances covered at different velocities (high or low intensity) [12,13]. However, absolute metrics such as these and the associated workload, are influenced by playing time. To combat this, and quantify match-play intensity, the workload relative to time played is typically calculated (e.g., relative distance; total distance divided by playing time; m·min^ − 1^). Previous research has shown that relative distance in forwards is statistically smaller than backs with forwards typically experiencing 64.6 to 66.8 m·min^ − 1^ and backs 71.1 to 73.3 m·min^ − 1^ [14,15], and this is likely due to the different positional roles of forward and backs. Regardless of position however, it is known that relative distance underestimates the true peak intensity of team sport match running [16,17]. This is because relative distance includes stoppages in running play (e.g., penalties, scrums), and low intensity movement (e.g., standing, walking or jogging). Instead, it is now recognised that a moving average approach is the most effective method of determining true peak running intensity, over shorter time durations than relative distance (i.e., 1- to 10-minute intervals). This has been shown in rugby union at the international level [10], in Australian Super Rugby [18,19], and English academy level [20]. At all three of these playing levels, the true peak running intensities exceed those of typical relative distances with 1-minute peaks from 154 m·min^ − 1^ to 185 m·min^ − 1^ (position dependent), and 10-minute peaks from 79 m·min^ − 1^ to 97 m·min^ − 1^ (position dependent) observed. Additionally, these data were presented for separate position groups within rugby union, however no study to date has differentiated 1- to 10-minute peak running data for whole, starters or substitute players. In team sport training activities are commonly prescribed in one minute increases in time (e.g., 5-minute activity) [20], and high-performance coaches may use peak running intensity intervals from matches to inform training drills to equate these intensities. This is most applicable and feasible for sport-specific drills such as small sided games, as these best replicate match conditions (i.e., physically and mentally) within training [11]. It is not suggested that practitioners use peak match running intensities to inform conditioning drills, for example, intervals, repeated sprints or linear speed training due to contextual differences between matches and these training modes [11]. Caution may also be suggested when using peak running intensity intervals from matches to inform training drills, as it does not consider internal load differences between matches and training contexts [11,21]. Aside from use for training prescription, quantifying peak running intensity via relative distance or moving average (i.e., 1- to 10-minute periods) can assist high performance staff in prescribing recovery post-match. More ‘intense’ matches with higher m·min^ − 1^ values across periods potentially requiring greater recovery time than those matches with lower values, from a running perspective. Additionally, match running performance may also be quantified by the duration that players are required to maintain high running intensities, for example, total distance covered in high intensity running (i.e., >  5.0 m·s^ − 1^) [12]. Matches with more time spent close to peak running intensity, as determined by moving average may also require larger recovery times, as players with more time close to individual peak running intensities will have expended more energy to maintain this running intensity. Moreover, the time spent close to the match peak for each player can indicate whole-match intensity, as opposed to isolated 1- to 10-minute peak periods. This concept has been shown in professional rugby league with time spent above 80%, 85%, and 90% of peak 1-minute moving average running intensity for acceleration data [22], however, has not been shown in rugby union, or with velocity data to date.

The purpose of this study was to build upon the limited prior work in rugby union regarding true peak running intensity, and quantify peak running intensity in professional rugby union across position groups, and peak running intensity differences between bout types (i.e., whole, starter, substitute) by describing 1- to 10-minute peak periods in English Premiership rugby velocity data, across positions. The purpose of this study was also to be the first to quantify the time spent above 80% and 90% of 1-minute peak match and season running intensity across position groups, and the first to quantify peak running intensity differences between bout types (i.e., whole match, starting and substitute). It was hypothesised that 1) backs position groups would demonstrate higher peak match running intensities than forwards positions groups in traditional relative distance and 1- to 10-minute peak period distances, and 2) that substitute players will demonstrate higher 1- to 10-minute peak period distances, than starting players or whole game players.

## Materials and methods

### Participants

Thirty-six professional rugby union players from one club participated in this study (descriptive data: Table 1). Players were included if they played at least four of the twenty-four matches (20%) in the 2022–23 Gallagher Premiership Rugby season (recruitment period: 01/09/2022 to 07/05/2023). Additionally, matches were only analysed if the player played at least 10-minutes within the match. Players were categorised into playing position groups: Forwards (*n* =  22) comprised of Tight 5 (*n* =  14; props, hookers, locks) and Backrow (*n* =  12; flankers and number 8); and Backs (*n* =  14) comprised of Half-backs (*n* =  4; scrum-half and fly-half) and Outside Backs (*n* =  10; inside centre, outside centre, wings and fullback). Players were also categorised into one of three bout type groups for each match, depending on their match involvement: 1) whole, 2) starter (i.e., is in the starting team but does not play the whole game), and 3) substitute. This study was approved by the Durham University Sport and Exercise Sciences and National Health Service Ethics Committees (North East Tyne and Wear South Research Ethics, Reference Number: 2/NE/0036, IRAS reference ID: 308072), and written informed consent was provided by each participant prior to participation.

**Table 1:** Participant information.

	*n*	Games Played	Age (Yrs)	Height (m)	Mass (kg)
**All**	36	12 ± 5	27.9 ± 4.3	1.87 ± 0.08	106.3 ± 14.3
**Forwards**	22	11 ± 6	27.6 ± 4.7	1.91 ± 0.06	116.3 ± 7.2
*Tight 5*	13	12 ± 6	28.9 ± 3.9	1.92 ± 0.07	118.8 ± 6.4
*Backrow*	9	8 ± 5	25.8 ± 5.3	1.90 ± 0.03	112.6 ± 6.8
**Backs**	14	13 ± 5	28.5 ± 3.8	1.80 ± 0.05	90.6 ± 5.7
*Half-backs*	4	16 ± 3	30.4 ± 3.2	1.76 ± 0.04	86.8 ± 5.9
*Outside Backs*	10	12 ± 5	27.7 ± 3.9	1.82 ± 0.04	92.2 ± 5.1

*n*, Number; Yrs, Years; m, Metres; kg, Kilograms.

### Procedures

The match activity of each participant was recorded via portable GPS unit (Catapult, Vector S7; 10 Hz), fitted into the built-in pouch in their playing jersey, or the built-in pouch of a GPS vest (Catapult, Vector Elite 2.1) worn directly underneath their playing jersey, depending on player preference. After each match, the data from each GPS unit were downloaded using the manufacturers proprietary software (Catapult Openfield). Data were then trimmed for each participants involvement within the match, and total playing time and relative distance (total distance divided by time; m·min^ − 1^) were calculated within the propriety software and exported for each participant. The entire raw velocity trace (m·s^ − 1^) was exported for each player. A custom script in R software (version 4.1.2, R Foundation for Statistical Computing) was used to convert the raw velocity trace from m·s^ − 1^ to m·min^ − 1^, and extract the 1- to 10-minute peak intensity periods via moving average (in line with previous research [10,18–20]), and calculate time spent above 80% and above 90% within the match of the peak 1-minute period value from the same match (sum of time above 80% of 1-minute match peak within current matches velocity m·min^ − 1^ trace, Time Match 80; sum of time above 90% of 1-minute match peak within current matches velocity m·min^ − 1^ trace, Time Match 90) (in line with previous research [22]), and time in that match spent above 80% and above 90% of the players season best 1-minute period (sum of time above 80% of season best 1-minute match peak within current matches velocity m·min^ − 1^ trace, Time Season 80; sum of time above 80% of season best 1-minute match peak within current matches velocity m·min^ − 1^ trace; Time Season 90). Data were then exported to Microsoft Excel where participant positions and bout type were added for sorting.

### Statistical analysis

Linear mixed models were performed in SPSS (v29, IBM, NY, USA) and used to compare the dependent variables (time played, relative distance, 1- to 10-minute peak intensity periods, time spend above 80% and above 90% match peak 1-minute period, time spend above 80% and above 90% season 1-minute period) between position groups, and in a second analysis to compare the dependent variables between bout type in Forwards and Backs groups. Fixed effects were position group (six levels), and bout type (three levels). Participant ID was included as a random effect in each model. Residual plots were used to assess normality, homoscedasticity, and independence of residuals. Pairwise comparisons for significant main effects were made based on estimated marginal means using least significant difference post hoc tests (α =  0.10; 90% confidence intervals). Cohen’s *d* effect sizes (ES) and 90% confidence intervals were calculated and considered *trivial*, < 0.20; *small*, 0.2 to 0.6; *moderate*, 0.6 to 1.2; *large*, 1.2 to 2.0; and *very large*, < 2.0 [23]. To further assess the practical relevance of the outcomes, magnitude base inferences (MBI) were also calculated (in line with previous research [10,20]) using the *p* values from the least significant difference post hoc tests of the linear mixed model, via a spreadsheet [24]. The smallest worthwhile difference was determined as 0.2 times the between-subject standard deviation of the measure, and assessed qualitatively as *most unlikely*, < 0.5%; *very unlikely*, 0.5 to 5%; *unlikely*, 5 to 25%; *possibly*, 25 to 75%; *likely*, 75 to 95%; *very likely*, 95 to 99.5%; and *most likely*, > 99.5%. Where confidence intervals crossed both of the thresholds for substantiveness (i.e., the effect could be substantially positive and negative), the difference was reported as *unclear* [25].

## Results

Raw data for each dependent variable are presented in Table 2, and online repository [26]. The results of the between position groups analysis is presented in Table 3. In time played, Tight 5 demonstrated *moderately* lower time played than the Outside Backs (*possibly moderate* ES: − 0.86 to − 0.45), and Half-backs demonstrated *moderately* lower time played than Outside Backs (*likely trivial moderate* ES: − 0.95 to − 0.42). All other group comparisons in time played demonstrated *trivial* or *small* differences. In relative distance, all group comparisons demonstrated *moderate* to *large* differences, except for the Tight 5 with a *small* lower relative distance than Backrow (*likely trivial small* ES: − 0.79 to − 0.32). All position groups showed *most likely moderate* to *most likely large* differences in 1- to 10-minute peak intensity periods, except for *small* lower m·min^ − 1^ in Tight 5 than Backrow (*possibly trivial small* and *possibly small*), and *trivially larger* m·min^ − 1^ in Half-backs than Outside Backs (*very likely trivial small* to *likely trivial small*). No position group comparison for Time Match 80, Time Match 90, Time Season 80 or Time Season 90 resulted in *moderate* or greater effect sizes, only *trivial* or *small* differences.

**Table 2:** Raw data.

Position	Samples (*n*)	Time Played (Min)	Rel. Dist. (m·min^ − 1^)	Peak Intensity Periods (m·min^ − 1^)										Time Match 80 (Min)	Time Match 90 (Min)	Time Season 80 (Min)	Time Season 90 (Min)
				1 Min	2 Min	3 Min	4 Min	5 Min	6 Min	7 Min	8 Min	9 Min	10 Min				
**Forwards**	233	61.8 ± 26.5	70 ± 6	159 ± 19	128 ± 15	113 ± 12	106 ± 12	100 ± 11	95 ± 10	92 ± 10	89 ± 10	87 ± 10	85 ± 10	10.2 ± 4.1	8.8 ± 3.6	8.0 ± 3.3	6.4 ± 2.7
*Whole*	74	89.9 ± 8.6	70 ± 6	164 ± 16	133 ± 14	119 ± 10	111 ± 12	104 ± 10	100 ± 9	96 ± 9	93 ± 9	91 ± 9	89 ± 8	14.1 ± 2.0	12.2 ± 1.8	11.0 ± 2.0	8.7 ± 1.7
*Starters*	87	63.3 ± 14.8	70 ± 6	157 ± 17	128 ± 15	113 ± 10	106 ± 11	101 ± 9	96 ± 8	93 ± 8	90 ± 8	88 ± 8	87 ± 7	10.6 ± 2.6	9.2 ± 2.3	8.3 ± 2.4	6.6 ± 2.0
*Substitutes*	72	30.9 ± 12.7	70 ± 7	154 ± 21	123 ± 15	108 ± 14	101 ± 12	95 ± 12	90 ± 11	87 ± 12	84 ± 11	82 ± 11	80 ± 11	5.6 ± 2.3	4.9 ± 2.0	4.6 ± 1.9	3.7 ± 1.5
**Tight 5**	157	59.7 ± 26.6	69 ± 6	156 ± 18	126 ± 15	112 ± 12	104 ± 12	98 ± 11	94 ± 10	91 ± 10	88 ± 10	86 ± 10	84 ± 10	10.0 ± 4.2	8.8 ± 3.6	7.9 ± 3.2	6.3 ± 2.6
**Backrow**	76	66.0 ± 25.9	72 ± 6	165 ± 18	132 ± 15	117 ± 12	110 ± 12	103 ± 10	99 ± 9	95 ± 9	92 ± 9	90 ± 9	88 ± 8	10.4 ± 4.0	9.0 ± 3.4	8.3 ± 3.5	6.7 ± 2.9
**Backs**	183	70.3 ± 26.6	78 ± 8	188 ± 19	150 ± 16	130 ± 15	121 ± 13	114 ± 12	109 ± 11	105 ± 10	102 ± 10	99 ± 10	97 ± 10	10.4 ± 3.5	8.7 ± 2.9	8.5 ± 3.0	6.7 ± 2.5
*Whole*	86	91.9 ± 6.2	76 ± 6	191 ± 17	150 ± 15	130 ± 13	121 ± 12	113 ± 11	108 ± 10	104 ± 9	101 ± 9	99 ± 8	96 ± 8	12.6 ± 2.1	10.5 ± 1.9	10.4 ± 1.8	8.2 ± 1.5
*Starters*	54	67.2 ± 13.5	79 ± 7	191 ± 21	152 ± 17	133 ± 15	123 ± 13	116 ± 12	112 ± 12	107 ± 11	103 ± 10	101 ± 11	99 ± 11	10.4 ± 2.4	8.7 ± 2.1	8.6 ± 2.3	6.8 ± 2.0
*Substitutes*	43	31.1 ± 14.8	83 ± 10	180 ± 21	146 ± 18	127 ± 16	119 ± 15	113 ± 14	107 ± 12	104 ± 12	101 ± 12	98 ± 13	96 ± 12	5.8 ± 2.2	5.0 ± 1.9	4.6 ± 1.9	3.6 ± 1.5
**Half-backs**	63	58.9 ± 28.3	82 ± 9	192 ± 19	154 ± 17	134 ± 15	126 ± 14	118 ± 14	113 ± 12	108 ± 12	104 ± 12	102 ± 12	100 ± 12	9.6 ± 4.0	8.0 ± 3.3	7.7 ± 3.4	6.0 ± 2.7
**Outside Backs**	120	76.3 ± 23.7	76 ± 7	186 ± 20	147 ± 16	128 ± 14	119 ± 12	112 ± 11	107 ± 10	104 ± 9	100 ± 9	98 ± 9	96 ± 8	10.8 ± 3.2	9.0 ± 2.7	8.9 ± 2.8	7.0 ± 2.2

All data are presented as mean ±  standard deviation. n, Number; m, Metres; Min, Minutes; Rel. Dist., Relative Distance.

**Table 3:** Between position group statistical analysis results.

Comparison	Time Played (Min)	Rel. Dist. (m·min^ − 1^)	Peak Intensity Periods (m·min^ − 1^)										Time Match 80 (Min)	Time Match 90 (Min)	Time Season 80 (Min)	Time Season 90 (Min)
			1 Min	2 Min	3 Min	4 Min	5 Min	6 Min	7 Min	8 Min	9 Min	10 Min				
**Forwards vs Backs**																
Mean Difference (±90%CI)	−6.8 (−17.4, 3.8)	−8.0 (−10.6, − 5.5)	−29.0 (−34.9, − 23.1)	−20.9 (−25.4, − 16.3)	−16.3 (−20.4, 12.1)	−14.8 (−18.9, − 10.8)	−13.8 (−17.4, − 10.1)	−13.1 (−16.6, − 9.6)	−12.4 (−15.7, − 9.1)	−12.6 (−15.8, − 9.5)	−11.7 (−15.0, − 8.4)	−11.1 (−14.3, − 7.9)	0.05 (−1.4, 1.5)	0.35 (−0.8, 1.5)	−0.11 (−1.3, 1.1)	0.07 (−0.9, 1.0)
ES (±90%CI)	−0.32 (−0.49, − 0.16); *Small*	−1.14 (−1.32, − 0.97); *Large*	−1.57 (−1.75, − 1.38); *Large*	−1.37 (−1.54, − 1.18); *Large*	−1.26 (−1.44, − 1.08); *Large*	−1.19 (−1.37, − 1.02); *Large*	−1.22 (−1.39, − 1.04); *Large*	−1.28 (−1.45, − 1.10); *Large*	−1.26 (−1.43, − 1.08); *Large*	−1.30 (−1.47, − 1.12); Large	−1.19 (−1.37, − 1.02); *Moderate*	−1.22 (-1.39, − 1.04); *Large*	−0.05 (−0.21, 0.12); *Trivial*	0.05 (−0.11, 0.21); *Trivial*	−0.15 (−0.32, 0.01); *Trivial*	-0.12 (−0.28, 0.05); *Trivial*
Qualitative Descriptor	*Likely Trivial*	*Most Likely*	*Most Likely*	*Most Likely*	*Most Likely*	*Most Likely*	*Most Likely*	*Most Likely*	*Most Likely*	*Most Likely*	*Most Likely*	*Most Likely*	*Likely Trivial*	*Likely Trivial*	*Likely Trivial*	*Likely Trivial*
**Tight 5 vs Backrow**																
Mean Difference (±90%CI)	−0.9 (−7.4, 5.6)	−0.6 (−2.4, 1.2)	−2.9 (−8.0, 2.2)	−1.9 (−6.1, 2.3)	−1.7 (−5.2, 1.9)	−1.6 (−4.9, 1.7)	−1.3 (−4.4, 1.7)	−1.2 (−4.0, 1.5)	−1.2 (−3.9, 1.5)	−1.2 (−3.8, 1.5)	−1.2 (−3.9, 1.5)	−1.2 (−3.7, 1.4)	−0.09 (−1.1, 0.9)	−0.06 (−0.9, 0.8)	−0.13 (−1.0, 0.7)	0.11 (−0.8, 0.6)
ES (±90 CI)	−0.24 (−0.47, − 0.01); *Small*	−0.56 (−0.79, − 0.32); *Small*	−0.51 (−0.74, − 0.27); *Small*	−0.38 (−0.61, − 0.15); *Small*	−0.47 (−0.71, − 0.24); *Small*	−0.47 (−0.70, − 0.23); *Small*	−0.43 (−0.66, − 0.20); *Small*	−0.49 (−0.72, − 0.25); *Small*	−0.47 (−0.70, − 0.23); *Small*	−0.42 (−0.65, − 0.19); *Small*	−0.45 (−0.68, − 0.21); *Small*	−0.51 (−0.75, − 0.28); *Small*	−0.09 (−0.32, 0.14); *Trivial*	−0.05 (−0.28, 0.18); *Trivial*	−0.14 (-0.37, 0.09); *Trivial*	-0.14 (-0.37, 0.09); *Trivial*
Qualitative Descriptor	*Likely Trivial*	*Likely Trivial*	*Possibly*	*Possibly Trivial*	*Possibly*	*Possibly*	*Possibly Trivial*	*Possibly Trivial*	*Possibly Trivial*	*Possibly Trivial*	*Possibly Trivial*	*Possibly*	*Unclear*	*Unclear*	*Likely Trivial*	*Likely Trivial*
**Tight 5 vs Half-backs**																
Mean Difference (±90%CI)	−6.0 (−17.9, 5.6)	−8.6 (−11.6, − 5.6)	−30.4 (−37.7, − 23.1)	−22.3 (−28.0, − 16.5)	−17.3 (−22.5, − 12.2)	−16.0 (−20.9, − 11.1)	−14.8 (−19.2, − 10.3)	−14.0 (−18.2, − 9.8)	−13.1 (−17.1, − 9.1)	−13.3 (−17.2, − 9.5)	−12.5 (−16.5, − 8.5)	−11.8 (−15.7, − 8.0)	0.12 (−1.5, 1.8)	0.41 (−1.0, 1.8)	−0.10 (−1.4, 1.3)	0.10 (−1.0, 1.2)
ES (±90 CI)	0.03 (−0.22, 0.28); *Trivial*	−1.81 (−2.08, − 1.52); *Large*	−2.00 (−2.28, − 1.70); *Large*	−1.80 (−2.07, − 1.51); *Large*	−1.72 (−1.99, − 1.43); *Large*	−1.70 (−1.97, − 1.41); *Large*	−1.61 −1.88, − 1.33); *Large*	−1.73 (−2.00, − 1.44); *Large*	−1.57 (−1.84, − 1.29); *Large*	−1.59 (−1.86, − 1.31); *Large*	−1.50 (−1.77, − 1.22); *Large*	−1.55 (−1.82, − 1.27); *Large*	0.11 (−0.14, 0.35); *Trivial*	0.21 (−0.04, 0.45); *Trivial*	0.05 (−0.19, 0.30); *Trivial*	0.09 (−0.16, 0.33); *Trivial*
Qualitative Descriptor	*Possibly*	*Most Likely*	*Most Likely*	*Most Likely*	*Most Likely*	*Most Likely*	*Most Likely*	*Most Likely*	*Most Likely*	*Most Likely*	*Most Likely*	*Most Likely*	*Unclear*	*Likely Trivial*	*Unclear*	*Unclear*
**Tight 5 vs Outside Backs**																
Mean Difference (±90%CI)	−7.6 (−18.6, 3.5)	−8.1 (−10.8, − 5.3)	−29.7 (−36.2, − 23.3)	−21.1 (−26.1, − 16.1)	−16.5 (−21.1, − 12.2)	−15.0 (−19.4, − 10.6)	−13.9 (−17.9, − 10.0	−13.3 (−17.1, − 9.5)	−12.7 (−16.2, − 9.1)	−12.8 (−16.2, − 9.4)	−11.9 (−15.4, − 8.4)	−11.3 (−14.8, − 7.8)	−0.03 (−1.5, 1.5)	0.29 (−1.0, 1.6)	−0.20 (−1.5, 1.1)	0.002 (−1.0, 1.0)
ES (±90 CI)	−0.66 (−0.86, − 0.45); *Moderate*	−1.16 (−1.37, − 0.94); *Moderate*	−1.65 (−1.87, − 1.41); *Large*	−1.39 (−1.61, − 1.17); *Large*	−1.30 (−1.51, − 1.07); *Large*	−1.21 (−1.42, − 0.99); *Large*	−1.23 (−1.44, − 1.01); *Large*	−1.29 (−1.51, − 1.07); *Large*	−1.32 (−1.54, − 1.10); *Large*	−1.34 (−1.55, − 1.11); *Large*	−1.24 (−1.46, − 1.02); *Large*	−1.29 (−1.51, − 1.07); *Large*	−0.19 (−0.38, 0.02); *Trivial*	−0.07 (−0.27, 0.13); *Trivial*	−0.35 (−0.55, − 0.15); *Small*	−0.32 (−0.52, − 0.12); *Small*
Qualitative Descriptor	*Possibly*	*Most Likely*	*Most Likely*	*Most Likely*	*Most Likely*	*Most Likely*	*Most Likely*	*Most Likely*	*Most Likely*	*Most Likely*	*Most Likely*	*Most Likely*	*Unclear*	*Likely Trivial*	*Likely Trivial*	*Unclear*
**Backrow vs Half-backs**																
Mean Difference (±90%CI)	−5.1 (−17.5, 7.2)	−8.0 (−11.1, − 4.9)	−27.5 (−35.3, − 19.7)	−20.4 (−26.6, − 14.2)	−15.7 (−21.1, − 10.2)	−14.4 (−19.6, − 9.2)	−13.5 (−18.2, − 8.7)	−12.8 (−17.2, − 8.3)	−11.9 (−16.2, − 9.4)	−12.1 (−16.2, − 8.1)	−11.3 (−15.5, − 7.0)	−10.7 (−14.7, − 6.6)	0.21 (−1.5, 1.9)	0.47 (−1.0, 1.9)	0.07 (−1.4, 1.5)	0.21 (−1.0, 1.4)
ES (±90 CI)	0.26 (−0.02, 0.54); *Small*	−1.22 (−1.52, − 0.91); *Large*	−1.47 (−1.78, − 1.15); *Large*	−1.35 (−1.65, − 1.03); *Large*	−1.24 (−1.54, − 0.93); *Large*	−1.23 (−1.53, − 0.92); *Large*	−1.25 (−1.55, − 0.94); *Large*	−1.32 (−1.62, − 1.00); *Large*	−1.18 (−1.47, − 0.87); *Moderate*	−1.27 (−1.56, − 0.95); *Large*	−1.14 (−1.44, − 0.84); *Moderate*	−1.13 (−1.43, − 0.82); *Moderate*	0.21 (−0.08, 0.49); *Small*	0.28 (−0.01, 0.56); *Small*	0.18 (−0.10, 0.46); *Trivial*	0.22 (−0.06, 0.50); *Small*
Qualitative Descriptor	*Possibly*	*Most Likely*	*Most Likely*	*Most Likely*	*Most Likely*	*Most Likely*	*Most Likely*	*Most Likely*	*Most Likely*	*Most Likely*	*Most Likely*	*Most Likely*	*Unclear*	*Possibly*	*Unclear*	*Unclear*
**Backrow vs Outside Backs**																
Mean Difference (±90%CI)	−6.7 (−18.3, 4.9)	−7.5 (−10.4, − 4.6)	−26.8 (−33.8, − 19.8)	−19.2 (−24.7, − 13.7)	−14.8 (−19.7, − 9.9)	−13.4 (−18.1, − 8.7)	−12.6 (−16.9, − 8.3)	−12.1 (−16.1, − 8.0)	−11.4 (−15.3, − 7.6)	−11.6 (−15.3, − 8.0)	−10.7 (−14.5, − 6.9)	−10.1 (−13.8, − 6.4)	0.10 (−1.5, 1.7)	0.35 (−1.0, 1.7)	−0.10 (−1.4, 1.3)	0.11 (−1.0, 1.2)
ES (±90 CI)	−0.42 (−0.66, − 0.18); *Small*	−0.61 (−0.85, − 0.36); *Moderate*	−1.13 (−1.39, − 0.87); *Moderate*	−0.98 (−1.23, − 0.72); *Moderate*	−0.84 (−1.09, − 0.58); *Moderate*	−0.75 (−0.99, − 0.49); *Moderate*	−0.86 (−1.10, − 0.60); *Moderate*	−0.86 (−1.11, − 0.61); *Moderate*	−0.91 (−1.16, − 0.66); *Moderate*	−1.00 (−1.25, − 0.74); *Moderate*	−0.87 (−1.12, − 0.62); *Moderate*	−0.85 (−1.10, − 0.59); *Moderate*	−0.09 (−0.33, 0.15); *Trivial*	−0.02 (−0.26, 0.22); *Trivial*	−0.20 (−0.44, 0.04); *Trivial*	−0.16 (−0.40, 0.09); *Trivial*
Qualitative Descriptor	*Possibly*	*Most Likely*	*Most Likely*	*Most Likely*	*Most Likely*	*Most Likely*	*Most Likely*	*Most Likely*	*Most Likely*	*Most Likely*	*Most Likely*	*Most Likely*	*Unclear*	*Likely Trivial*	*Unclear*	*Unclear*
**Half-backs vs Outside Backs**																
Mean Difference (±90%CI)	−1.6 (−8.8, 5.7)	0.5 (−1.5, 2.6)	0.7 (−5.1, 6.4)	1.2 (−3.6, 6.0)	0.8 (−3.2, 4.8)	1.0 (−2.8, 4.8)	0.9 (−2.6, 4.3)	0.7 (−2.4, 3.8)	0.5 (−2.6, 3.5)	0.5 (−2.5, 3.5)	0.6 (−2.5, 3.6)	0.5 (−2.3, 3.4)	−0.14 (−1.3, 1.0)	−0.13 (−1.1, 0.8)	−0.14 (−1.1, 0.8)	−0.10 (−0.9, 0.7)
ES (±90 CI)	−0.69 (−0.95, − 0.42); *Moderate*	0.69 (0.42, 0.95); *Moderate*	0.29 (0.03, 0.55); *Small*	0.39 (0.13, 0.65); *Small*	0.39 (0.13, 0.65); *Small*	0.54 (0.27, 0.79); *Small*	0.48 (0.22, 0.74); *Small*	0.52 (0.26, 0.77); *Small*	0.38 (0.12, 0.63); *Small*	0.39 (0.13, 0.65); *Small*	0.42 (0.16, 0.68); *Small*	0.43 (0.17, 0.69); *Small*	−0.33 (−0.58, − 0.07); *Small*	−0.34 (−0.59, − 0.08); *Small*	−0.42 (−0.67, − 0.16); *Small*	−0.42 (−0.67, − 0.16); *Small*
Qualitative Descriptor	*Likely Trivial*	*Likely Trivial*	*Very Likely Trivial*	*Likely Trivial*	*Likely Trivial*	*Likely Trivial*	*Likely Trivial*	*Likely Trivial*	*Likely Trivial*	*Likely Trivial*	*Likely Trivial*	*Likely Trivial*	*Very Likely Trivial*	*Very Likely Trivial*	*Very Likely Trivial*	*Very Likely Trivial*

m, metres; Min, Minutes; Rel. Dist., Relative Distance; m, Meters; ES, Effect Size; CI, Confidence Interval.

The results of the between bout type analysis are presented in Table 4. In time played, all bout type comparisons for Forwards and Backs resulted in *most likely very large* differences. In relative distance, all bout type comparisons in Forwards resulted in *trivial* differences. In Backs *small* differences were observed in Whole vs Starters and Starters vs Substitute comparisons, however *moderately larger* m·min^ − 1^ was shown by Substitutes than Whole (*likely moderate* ES: − 1.29 to − 0.64). In 1- to 10-minute peak intensity periods, all bout type comparisons in Forwards and Backs resulted in *trivial* to *small* differences, except for 1) the *moderately larger* m·min^ − 1^ shown by Forwards Whole than Forward Substitutes in 2- to 10-minute peak periods (*possibly moderate* ES: 0.41 to 0.97, to *most likely moderate* ES: 0.69 to 1.27), 2) *moderately larger* m·min^ − 1^ shown by Forwards Starters than Forward Substitutes in the 10-minute peak intensity period (*most likely moderate* ES: 0.47 to 1.01), and 3) *moderately larger* m·min^ − 1^ shown by Backs Whole than Backs Substitutes in the 1-minute peak intensity period (*most likely moderate* ES: 0.29 to 0.91). For Time Match 80, Time Match 90, Time Season 80 or Time Season 90 all bout type comparisons for Forwards and Backs resulted in *most likely moderate*, *most likely large*, or *most likely very large* differences.

**Table 4:** Between bout type statistical analysis results.

Comparison	Time Played	Rel. Dist. (m·min^ − 1^)	Peak Intensity Periods (m·min^ − 1^)										Time Match 80 (Min)	Time Match 90 (Min)	Time Season 80 (Min)	Time Season 90 (Min)
			1 Min	2 Min	3 Min	4 Min	5 Min	6 Min	7 Min	8 Min	9 Min	10 Min				
**Forwards**																
***Whole vs Starters***																
Mean Difference (±90%CI)	26.8 (23.4, 30.2)	−2.0 (−3.8, − 0.1)	3.0 (−2.2, 8.2)	2.1 (−2.0, 6.3)	1.6 (−1.7, 4.9)	1.4 (−2.0, 4.7)	1.4 (−1.6, 4.3)	1.9 (−0.8, 4.6)	1.3 (−1.4, 4.0)	1.0 (−1.6, 3.7)	0.7 (−2.0, 3.4)	0.5 (−2.0, 3.0)	3.5 (2.8, 4.1)	2.9 (2.4, 3.5)	2.7 (2.1, 3.3)	2.1 (1.6, 2.6)
ES (±90%CI)	2.14 (1.81, 2.46); *Very Large*	0.10 (−0.16, 0.36); *Trivial*	0.40 (0.14, 0.66); *Small*	0.37 (0.11, 0.64); *Small*	0.53 (0.26, 0.79); *Small*	0.39 (0.13, 0.65); *Small*	0.40 (0.13, 0.66); *Small*	0.48 (0.21, 0.74); *Small*	0.42 (0.15, 0.68); *Small*	0.39 (0.13, 0.65); *Small*	0.34 (0.08, 0.60); *Small*	0.34 (0.08, 0.60); *Small*	1.51 (1.20, 1.79); *Large*	1.44 (1.14, 1.72); *Large*	1.19 (0.90, 1.46); *Moderate*	1.08 (0.80, 1.36); *Moderate*
Qual. Descriptor	*Most Likely*	*Possibly*	*Possibly*	*Possibly*	*Possibly*	*Possibly*	*Possibly*	*Possibly*	*Possibly*	*Possibly*	*Likely Trivial*	*Possibly*	*Most Likely*	*Most Likely*	*Most Likely*	*Most Likely*
***Whole vs Substitute***																
Mean Difference (±90%CI)	58.7 (55.1, 62.4)	−1.8 (−3.8, 0.3)	6.4 (0.6, 12.1)	7.3 (2.7, 11.9)	7.5 (3.9, 11.2)	7.7 (4.0, 11.4)	7.5 (4.2, 10.8)	7.6 (4.6, 10.6)	7.4 (4.4, 10.4)	6.8 (3.9, 9.7)	6.5 (3.5, 9.6)	7.5 (4.7, 10.3)	8.3 (7.6, 9.1)	7.2 (6.6, 7.8)	6.5 (5.9, 7.2)	5.2 (4.6, 5.8)
ES (±90%CI)	5.43 (4.81, 5.99); Very Large	0.02 (−0.25, 0.30); *Trivial*	0.54 (0.26, 0.82); *Small*	0.69 (0.41, 0.97); *Moderate*	0.89 (0.60, 1.17); *Moderate*	0.85 (0.56, 1.12); *Moderate*	0.88 (0.59, 1.16); *Moderate*	0.93 (0.64, 1.21); *Moderate*	0.91 (0.62, 1.19); *Moderate*	0.89 (0.60, 1.17); *Moderate*	0.84 (0.55, 1.12); *Moderate*	0.99 (0.69, 1.27); *Moderate*	3.90 (3.41, 4.34); *Very Large*	3.80 (3.32, 4.23); *Very Large*	3.33 (2.89, 3.73); *Very Large*	3.04 (2.62, 3.42); *Very Large*
Qual. Descriptor	*Most Likely*	*Possibly*	*Possibly*	*Possibly*	*Very Likely*	*Very Likely*	*Very Likely*	*Most Likely*	*Most Likely*	*Most Likely*	*Very Likely*	*Most Likely*	*Most Likely*	*Most Likely*	*Most Likely*	*Most Likely*
***Starters vs Substitute***																
Mean Difference (±90%CI)	31.9 (28.6, 35.3)	0.2 (−1.5, 1.9)	3.4 (−1.5, 8.2)	5.2 (1.3, 9.1)	5.9 (2.9, 9.0)	6.3 (3.2, 9.4)	6.1 (3.4, 8.9)	5.7 (3.2, 8.2)	6.1 (3.6, 8.6)	5.8 (3.4, 8.2)	5.8 (3.3, 8.4)	7.0 (4.7, 9.4)	4.9 (4.2, 5.5)	4.2 (3.7, 4.8)	3.8 (3.3, 4.4)	3.1 (2.6, 3.5)
ES (±90%CI)	2.32 (1.97, 2.65); *Very Large*	−0.07 (−0.33, 0.19); *Trivial*	0.18 (−0.09, 0.44); *Trivial*	0.32 (0.05, 0.58); *Small*	0.46 (0.20, 0.73); *Small*	0.49 (0.22, 0.75); *Small*	0.57 (0.30, 0.84); *Small*	0.56 (0.29, 0.82); *Small*	0.58 (0.31, 0.84); *Small*	0.57 (0.30, 0.84); *Small*	0.58 (0.31, 0.84); *Small*	0.74 (0.47, 1.01); *Moderate*	2.00 (1.67, 2.31); *Very Large*	1.97 (1.64, 2.28); *Large*	1.73 (1.42, 2.03); *Large*	1.59 (1.28, 1.88); *Large*
Qual. Descriptor	*Most Likely*	Unclear	*Possibly*	*Likely*	*Very Likely*	*Very Likely*	*Very Likely*	*Very Likely*	*Most Likely*	*Most Likely*	*Very Likely*	*Most Likely*	*Most Likely*	*Most Likely*	*Most Likely*	*Most Likely*
**Backs**																
***Whole vs Starters***																
Mean Difference (±90%CI)	24.6 (21.4, 27.8)	−0.7 (−2.7, 1.2)	2.1 (−3.4, 7.5)	1.3 (−3.4, 6.0)	0.3 (−3.8, 4.4)	1.3 (−2.3, 4.9)	−0.7 (−4.1, 2.6)	−0.9 (−3.9, 2.1)	0.4 (−2.5, 3.3)	0.2 (−2.6, 3.1)	0.3 (−2.6, 3.2)	0.5 (−2.2, 3.2)	2.5 (1.9, 3.2)	2.0 (1.4, 2.6)	2.2 (1.6, 2.8)	1.7 (1.2, 2.2)
ES (±90%CI)	2.55 (2.16, 2.92); *Very Large*	−0.47 (−0.75, − 0.18); *Small*	0.01 (−0.27, 0.30); *Trivial*	−0.13 (−0.41, 0.16); *Trivial*	−0.21 (−0.50, 0.07); *Small*	−0.22 (−0.50, 0.07); *Small*	−0.31 (−0.60, − 0.03); *Small*	−0.36 (−0.64, − 0.07); *Small*	−0.24 (−0.52, 0.05); *Small*	−0.25 (−0.54, 0.04); *Small*	−0.26 (−0.54, 0.03); *Small*	−0.26 (−0.55, 0.03); *Small*	0.96 (0.65, 1.25); *Moderate*	0.86 (0.55, 1.15); *Moderate*	0.88 (0.58, 1.18); *Moderate*	0.75 (0.46, 1.05); *Moderate*
Qual. Descriptor	*Most Likely*	*Possibly*	*Possibly*	*Unclear*	*Unclear*	*Possibly*	*Unclear*	*Possibly*	*Unclear*	*Unclear*	*Unclear*	*Unclear*	*Most Likely*	*Most Likely*	*Most Likely*	*Most Likely*
***Whole vs Substitute***																
Mean Difference (±90%CI)	60.5 (57.0, 64.0)	−3.3 (−5.5, − 1.0)	15.9 (9.6, 22.1)	9.0 (3.6, 14.5)	9.1 (4.4, 13.9)	7.6 (3.4, 11.7)	4.9 (1.0, 8.8)	5.2 (1.7, 8.8)	4.8 (1.5, 8.2)	4.2 (0.9, 7.5)	3.7 (0.4, 7.1)	5.0 (1.9, 8.1)	7.1 (6.3, 7.8)	5.7 (5.0, 6.4)	6.5 (5.8, 7.1)	5.1 (4.6, 5.7)
ES (±90%CI)	6.15 (5.42, 6.81); *Very Large*	−0.97 (−1.29, − 0.64); *Moderate*	0.60 (0.29, 0.91); *Moderate*	0.26 (−0.05, 0.57); *Small*	0.25 (−0.06, 0.55); *Small*	0.10 (−0.21, 0.41); *Trivial*	−0.0003 (−0.31, 0.31); *Trivial*	0.07 (−0.24, 0.38); *Trivial*	0.07 (−0.24, 0.37); *Trivial*	0.02 (−0.29, 0.32); *Trivial*	0.005 (−0.30, 0.31); *Trivial*	0.10 (−0.21, 0.40); *Trivial*	3.15 (2.69, 3.57); *Very Large*	2.83 (2.39, 3.23); *Very Large*	3.23 (2.76, 3.66); *Very Large*	2.93 (2.48, 3.34); *Very Large*
Qual. Descriptor	*Most Likely*	*Likely*	*Most Likely*	*Very Likely*	*Very Likely*	*Very Likely*	*Likely*	*Likely*	*Likely*	*Likely*	*Likely*	*Likely*	*Most Likely*	*Most Likely*	*Most Likely*	*Most Likely*
***Starters vs Substitute***																
Mean Difference (±90%CI)	35.9 (32.1, 39.7)	−2.5 (−4.9, − 0.2)	13.8 (7.4, 20.2)	7.7 (2.1, 13.3)	8.8 (4.0, 13.7)	6.3 (2.0, 10.6)	5.6 (1.7, 9.6)	6.2 (2.6, 9.7)	4.5 (1.0, 7.9)	4.0 (0.6, 7.4)	3.4 (0.03, 6.8)	4.5 (1.3, 7.7)	4.6 (3.8, 5.3)	3.6 (3.0, 4.4)	4.3 (3.6, 5.0)	3.4 (2.9, 4.0)
ES (±90%CI)	2.56 (2.09, 2.99); *Very Large*	−0.49 (−0.83, − 0.14); *Small*	0.52 (0.17, 0.85); *Small*	0.35 (0.01, 0.68); *Small*	0.42 (0.08, 0.76); *Small*	0.29 (−0.05, 0.63); *Small*	0.28 (−0.06, 0.61); *Small*	0.37 (0.03, 0.71); *Small*	0.26 (−0.08, 0.59); *Small*	0.22 (−0.11, 0.56); *Small*	0.21 (−0.13, 0.54); *Small*	0.29 (−0.05, 0.63); *Small*	1.99 (1.57, 2.39); *Large*	1.82 (1.41, 2.20); *Large*	1.92 (1.50, 2.30); *Large*	1.79 (1.38, 2.17); *Large*
Qual. Descriptor	*Most Likely*	*Likely*	*Very Likely*	*Likely*	*Very Likely*	*Likely*	*Likely*	*Very Likely*	*Likely*	*Likely*	*Likely*	*Likely*	*Most Likely*	*Most Likely*	*Most Likely*	*Most Likely*

m, metres; Min, Minutes; Rel. Dist., Relative Distance; m, Meters; ES, Effect Size; CI, Confidence Interval.

## Discussion

The purpose of this study was to 1) describe 1- to 10-minute peak intensity periods in English Premiership rugby velocity data, 2) to quantify the time spent above 80% and 90% of 1-minute peak match running intensity across position groups, and 3) quantify peak running intensity differences between bout types. The results highlight that between positions, there are meaningful differences between forwards and backs position groups in peak running intensity, confirming the first hypothesis. The results also highlight that substitute players do not demonstrate meaningfully higher running intensities than whole or stating players, rejecting the second hypothesis. The main findings of this study were 1) backs position groups demonstrated *moderate* to *large* higher relative distances and 1- to 10-minute peak intensity period distances than forwards positions groups; 2) *moderate* or greater differences were not observed within forwards position groups, or within backs position groups for 1- to 10-minute peak intensity periods; 3) no position group comparison for Time Match 80, Time Match 90, Time Season 80 or Time Season 90 resulted in *moderate* or greater differences between groups; 4) substitute players demonstrated *moderately larger* relative distance only in backs whole vs backs substitute; 5) substitute players demonstrated lower distances in 1- to 10-minute peak intensity periods than whole or starters, although *moderate* differences were only observed when forwards substitutes were compared to forwards whole in 2- to 10-minute periods and to forward starters in the 10-minute period, and backs substitutes compared to backs whole in the 1-minute period; and 6) *large* to *very large* differences were observed in Time Match 80, Time Match 90, Time Season 80 or Time Season 90 with substitute players demonstrating lower time played than whole or stater players in both the forwards and backs. When training athletes to cope with the peak running demands of professional rugby union match-play, this study suggests that backs are required to perform higher running intensities within matches than forwards and should be trained accordingly. This study showed that *most likely moderate* to *most likely large* greater relative distances were performed by backs than forwards position groups. This was expected as previous works have demonstrated the same [14,15], however, the mean relative distances shown in this study are higher than those shown in previous studies (forwards: 70 vs 64.6–66.8 m·min^ − 1^; backs 78 vs 71.1–73.3 m·min^ − 1^). Additionally, *most likely moderate* to *most likely large* greater distances in 1- to 10-minute peak intensity periods were observed in backs position groups than forwards position groups (Table 3). Again, these results were expected as previous works has shown similar [10,20].

The differences in backs vs forwards position groups in running intensity may be explained by the tactical differences in each position’s role. It is well accepted that forwards are bigger and engage in more impacts but less running distance (including high speed) than backs [27–29]. It is also well accepted that backs run with higher speeds and more frequently than the forwards [1,4,27,28]. In contrast however, Peek et al. [18], did not show any meaningful difference between position groups in peak running intensity, due to a large prediction interval and individual player variation. Additionally, Delaney et al. [10], did not observe *moderate* effect size differences between the loose forwards group (i.e., backrow), and any other position group (i.e., tight 5, half-backs, and outside-backs). Whereas the results of the present study did demonstrate *moderate* or *large* differences between backrow and outside backs, and backrow and half-back groups across 1- to 10-minute peak intensity periods. These results could be due to the populations used, where Delaney et al. [10], used international level players, and the present study professional players; or apparent similarities to half-backs, and outside-backs in running ability by backrow players at the international level. Similarly, the results of the present study showed only *small* differences within position groups (i.e., tight 5 vs backrow and half-backs vs outside backs) in 1- to 10-minute intensity periods. These results are unexpected, again due to tactical differences between position groups indicating potential running intensity differences. However, this may be understood as the nature of peak 1- to 10-minute periods within a match are such that a large number of the team may need to run at a high intensity at the same time in response to match scenarios (e.g., performing or defending multi-phase attacks). Although the forwards differ from the backs, the running intensity demands of the forwards and backs are uniform within the groups, and differences within the forwards and backs position groups may rather be reflected in volume (e.g., total distance) rather than intensity (e.g., relative distance, and peak intensity periods) [30,31].

Only *trivial* or *small* differences were observed in Time Match 80, Time Match 90, Time Season 80 or Time Season 90 variables between position groups. This indicates that there are little to no practical differences in time spent within above 80% and 90% of individual match 1-minute peak period and above 80% and 90% of individual season best 1-minute peak period within matches across position groups, and this may be due to the individual nature of these metrics. Although there are differences in magnitude of 1-minute peak periods across positions (Table 2) this does not translate into differences in time spent above 80% and 90% of the match 1-minute peak intensity period due to players playing to within their individually set match 1-minute peak period. Also interesting is that even when *moderate* differences in time played are observed between positions (i.e., between tight 5 and outside backs, and half-backs and outside backs, *possibly* and *likely trivial* respectively), there are still only *trivial* or *small* differences in time spent above 80% and 90% of the match 1-minute peak intensity period. This could again be due to the individual nature of the metric.

This study found that substitute players demonstrated *moderately larger* relative distance only in backs whole vs backs substitutes. It was expected that substitute players would demonstrate meaningfully higher relative distances than whole or starter players in both forwards and backs, due to lower time played and less need to pace themselves [6,8]. Although, the results of the present study may be due to the nature of the relative distance variable and its relationship with time played; backs whole demonstrated the highest average playing time across the team (Table 2). Starting backs may also be more likely to finish the match, and not be substituted in comparison to forwards due to positional differences in roles (e.g., physicality, scum and ruck involvements). However, the results of the present study are both supported and contrasted by previous works. Tee et al. [8], showed *unclear* differences in relative distance in backs whole vs backs substitutes, *likely moderate* differences in backs whole vs backs starters, and similar results to the present study in forwards. The results of this paper however may not be directly comparable, due to Tee et al. [8]’s, data being drawn from the 2013 super rugby season from a South African team. Recent rugby union law changes, mean that comparisons with older studies should be done with caution. Additionally, in further support of the findings of the present study Lacome et al. [9], found *very likely small* differences in relative distance between forward whole and forward substitutes, and *likely small* differences between forward starters and forward substitutes, and *likely small* differences between backs starters and backs substitutes. However, in contrast to the present study’s findings also showed *very likely small* differences between backs whole and backs substitutes in relative distance. Again, the results of Lacome et al. [9], may be compared with some caution as they are from French international matches between 2005 and 2010, whereas the present study used data from one club during the 2022–23 Gallagher Premiership Rugby season. Lacome et al. [9], also did not use GPS, and instead used a multiple-camera computerised player-tracking system to quantify player locomotion

The most surprising result of this study was that substitute players demonstrated lower and not higher distances in 1- to 10-minute peak intensity periods than whole or starters. Although the majority of these were *small* effects, the second hypothesis was none-the-less rejected. The greatest differences (*possibly moderate* to *most likely moderate*) in peak intensity periods were observed between forwards substitutes and forwards whole in 2- to 10-minute periods and forward starters in the 10-minute period, and backs substitutes compared to backs whole in the 1-minute period. This may be a result of the best and most physically competitive 15 players being selected to start matches, and the remaining eight substitutes being used to replace the ‘better’ players once those starter players fatigue and are not able to perform at a standard greater than the substitute players can provide [5,6]. Anecdotally, substitute players run at higher intensities due to lower time played and less need to pace themselves [6,8], however, substitutes may also not achieve higher running intensities as they are exposed to less running scenarios within matches. I.e., starters and whole game players who have greater time played, have more opportunities and exposure to different running related scenarios within a match and multiple 1- to 10-minute periods where a maximum for each period is selected to represent the match. Although, our results demonstrate that substitute players play on average almost 40% of an 80-minute match (forwards: 30.9 ±  12.7 min; backs: 31.1 ±  14.8 min; Table 2), and whilst less time than whole game and starting players, may also be sufficient time to be exposed to intense periods of match play. Week to week, players may start matches or substitute, and therefore all players should be trained to be able to cope with the peak running intensities and time played demonstrated by whole game or starting players.

In contrast to the position analysis, in the bout type analysis *large* to *very large* differences were observed in Time Match 80, Time Match 90, Time Season 80 or Time Season 90 with substitute players demonstrating lower time played than whole or starter players in both the forwards and backs. This result is expected, as it is heavily influenced by the time played by each bout type, with *most likely very large* differences in time played between all bout comparisons and substitute players demonstrating the least time played. The longer players play for directly transfers to more opportunities to run above 80% and 90% of individual match and season 1-minute peaks. Although the results show that substitute players set lower match 1-minute peak distances than whole or starters (Table 2), which may be easier to run above 80% and 90% of, the substitutes are still restricted by less time on the field. The combination of less time played, lower 1- to 10-minute peak intensity period distances, and less time played above 80% and 90% of match and season 1-minute peaks, supports that matches are likely less fatiguing for substitutes than starters or whole game players. Less recovery and more volume within the training week may be required to account for this.

A potential limitation of the present study is that it is data from only one team in the English Premiership and therefore may not be generalisable to other teams within the English Premiership or other levels of rugby (i.e., international, semi-professional and amateur), or to other non-English professional competitions (e.g., Super Rugby Pacific, United Rugby Championship). The utilisation of only one team also meant a relatively small sample size of 36 participants. A larger sample size could strengthen the statistical analysis results, and generalisability of the results. Additionally, only male athletes were utilised, and therefore, these results may not be applicable to female rugby union athletes, or representative of professional women’s rugby competitions (e.g., Premiership Women’s Rugby, Women’s Elite Rugby). Future research is warranted across different populations and levels of rugby union population to quantify peak running intensity. Further research is also warranted to expand on the time played above 80% and 90% of match and season 1-minute peak variables, to better understand any potential effect on fatigue and recovery.

In conclusion, there are meaningful differences between position groups in peak running intensity in professional rugby union, and substitute players perform lower peak relative distance than whole or starters, as shown in one English Premiership team. This study builds upon previous work done in rugby union regarding true peak running intensity and is the first to quantify the time spent above 80% and 90% of 1-minute peak match running intensity across position groups, and the first to quantify peak running intensity differences between bout types. Practitioners may use the results of this study as normative data, to inform training prescription.

## Practical applications

When prescribing post-match recovery and quantifying the load experienced by players in a match, practitioners should consider both the running intensity (1–10 minute periods), and the time spent above 80% and 90% of 1-minute peak; with more intense or fatiguing matches indicated by higher m·min^ − 1^ values across periods and greater time spent above 80% and 90% of 1-minute peak. Sport scientists may also be able to analyse these data during a match, and feedback regarding player running performance and possible associated fatigue to tactical coaches to assist with substitution decisions. There are differences between rugby union forwards and backs in peak running intensity. When training athletes to cope with the peak running demands of match-play, backs are required to perform higher running intensities than forwards and should be trained accordingly. Substitute players do not demonstrate higher running intensities than whole or stating players. Week to week, players may start matches or substitute; all players should be trained to be able to cope with the peak running intensities demonstrated by whole game or starting players.
